# Herb-Induced Liver Injury—A Challenging Diagnosis

**DOI:** 10.3390/healthcare10020278

**Published:** 2022-01-31

**Authors:** David Ricardo da Conceição Marçal Alves Nunes, Cristina Sofia de Jesus Monteiro, Jorge Luiz dos Santos

**Affiliations:** 1 Faculdade de Ciências da Saúde, Universidade da Beira Interior, 6200-506 Covilhã, Portugal; csjmonteiro@sapo.pt (C.S.d.J.M.); jlsantos@fcsaude.ubi.pt (J.L.d.S.); 2 UFBI-Pharmacovigilance Unit of Beira Interior, Faculdade de Ciências da Saúde, Universidade da Beira Interior, 6200-506 Covilhã, Portugal; 3 CICS-UBI-Health Sciences Research Centre, Faculdade de Ciências da Saúde, Universidade da Beira Interior, 6200-506 Covilhã, Portugal

**Keywords:** hepatotoxicity, herbs, traditional medicine, causality assessment, herbal quality

## Abstract

Herb-induced liver injury (HILI) can be caused by supplements containing herbs, natural products, and products used in traditional medicine. Herbal products’ most common adverse reaction is hepatotoxicity. Almost every plant part can be used to make herbal products, and these products can come in many different forms, such as teas, powders, oils, creams, capsules, and injectables. HILI incidence and prevalence are hard to estimate and vary from study to study because of insufficient large-scale prospective studies. The diagnosis of HILI is a challenging process that requires not only insight but also a high degree of suspicion by the clinician. HILI presents with unspecific symptoms and is a diagnosis of exclusion. For diagnosis, it is necessary to make a causality assessment; the Council for International Organizations of Medical Sciences assessment is the preferred method worldwide. The most effective treatment is the suspension of the use of the suspected herbal product and close monitoring of liver function. The objective of this review is to highlight the necessary steps for the clinician to follow to reach a correct diagnosis of herb-induced liver injury. Further studies of HILI are needed to better understand its complexity and prevent increased morbidity and mortality.

## 1. Introduction

The liver plays a crucial role in maintaining body homeostasis [1] by performing several functions, such as the metabolization of different compounds, both endogenous, such as proteins [2,3], lipids [2,3], and carbohydrates [2,3], and exogenous, of which drugs are a prime example [4,5]. The liver is the main organ responsible for metabolism [5,6,7]; as such, it is at increased risk of injury induced by the formation of hepatotoxic metabolites during metabolic degradation [7].

Drug-induced liver injury (DILI) can be caused by drugs, food supplements, and herbal medicines [8,9]. The incidence of DILI is estimated between 14 and 19 cases per 100,000 population [10,11,12]. DILI has a high mortality rate of around 10% [5]. So far, more than 1000 different chemical compounds have been associated with DILI [7,13]. DILI is a hepatic injury of which the causative chemical agent is, for the most part, well characterized, whereas herb-induced liver injury (HILI) results from the harmful effect of an extract containing several different compounds, of which at least one is responsible for the hepatotoxic effect [14].

HILI can be caused by supplements containing herbs, natural products, and products used in traditional medicine all over the world [15,16,17,18,19]. As can be seen in Table 1, herbal products’ market share has seen an increase worldwide [16,18,20,21,22]. This trend may be attributable to several factors. Among them are the general population’s belief that herbal substances do not cause harm because they are natural [18,19,20,21], the claims of efficacy that have been made by advocates of this type of products, patients’ dissatisfaction with the results of modern medicine, the high costs and side effects of traditional medicines produced by the pharmaceutical industry, etc. [22]. Patients normally do not report to their doctors the use of natural products; as such, the clinician must inquire about their consumption on a regular basis [16,18,20,21]. Therapeutic claims regarding these products are, for the most part, unproven by scientific evidence. Nonetheless, their harms are real [18,21]. Herbal products contain unknown concentrations of several different compounds that have biological effects [18,20]. Hepatoxicity is the most common adverse reaction [20].

HILI presents with unspecific symptoms and is a diagnosis of exclusion. The diagnosis of HILI is a challenging process that requires not only insight but also a high degree of suspicion by the clinician. As such, the objective of this review is to highlight the necessary steps to be followed to reach a correct diagnosis of herb-induced liver injury.

## 2. Methods and Study Selection

We performed a nonsystematic (narrative) review of herb-induced liver injury using the following keywords on PubMed: Hepatotoxicity; DILI; HILI; Herbs; Traditional medicine; Causality assessment; Herbal quality. Studies were selected by authors based on title and abstract. The period of article selection was between 1 January 1981 and 1 November 2021. For this manuscript, we used 3 books and 2 websites written in Portuguese. The remaining references used for this manuscript were in English.

Because of the nature of this review, the authors eventually selected the papers according to their analytical criteria, and thus bias related to article selection and small sample size are important to be taken into account.

## 3. Traditional Medicine

Traditional medicine (TM) is defined by the World Health Organization as theories, beliefs, experiences, knowledge, skills, and practices that people of different indigenous cultures use to prevent, diagnose, treat, or maintain health, both physical and mental [21].

This type of medicine is increasingly being used by patients worldwide to prevent and treat disease [21,42,43,44]. This trend might have been brought to its current state because of globalization of not just goods, services, and people but cultural practices and health practitioners. As an example, TM with its origin in one region of the world can today be used anywhere. TM has brought both potential health benefits and side effects [21]. The herbs and herbal products described in Table 1 can be found all over the world and can be used by everyone.

Below are some examples of TMs that encourage the use of herbal products: Ayurvedic medicine, from the Himalayas and India; Sowa Rigpa, from Bhutan; herbal medicine from Bangladesh; Jamu, from Indonesia; Thai, from Thailand; Kampo, from Japan; Korean traditional medicine; traditional Chinese medicine; Unani medicine, from Perso-Arabic TM; African TM; Inca TM; Māori TM; Caribbean TM; Native American TM; and other Indigenous traditional medicine [15,18,19,21,42,45,46,47,48]. Some examples of these products can be seen in Table 1.

## 4. Herbal Products

Herbal products are made using plant parts and other materials, either alone or in combinations [21].

Liver injury can be caused by a multitude of different herbs and plant components [45]. Herbal products can be made of different raw materials, such as leaves, flowers, fruits, roots, seeds, bark, and the plant’s aerial part [20,45], as seen in Table 1. Finished herbal products can come in many different forms, such as teas, powders, oils, creams, capsules, and injectables [20].

## 5. Epidemiology

HILI has an incidence and prevalence that is hard to estimate for the general population because of a lack of large-scale prospective studies [19,20,49]. In Korea, both a nationwide multicenter study and a prospective study revealed a HILI incidence of 0.6% among hospitalized patients [16]. In Germany, a prospective hospital-based and a large-scale study of 21,470 patients revealed that 0.12% of hospitalized patients presented HILI [16], whereas the Spanish DILI registry revealed that in 2016, around 6% of cases were due to HILI [20].

Prevalence values of HILI are also variable. Prospective studies have revealed that in as much as in 71% of cases of DILI in Singapore and 73% of cases of DILI in Korea, some sort of herbal product was involved. In India, there is high consumption of herbs; nonetheless, prevalence data as low as 1.3% of cases of HILI among DILI cases have been reported [20,49].

The reason for this discrepancy in numbers may be studies with small sampling [19,49].

## 6. HILI Classification

Both DILI and HILI are classified as either intrinsic or idiosyncratic [15,20]. The importance of discriminating between these two types of injury resides in the fact that the intrinsic type is dose-dependent and has a short latency time, while idiosyncratic injury is not dose-dependent and has long latency time. This means that from a clinical perspective, it is easier to identify the etiological agent in intrinsic than in idiosyncratic injury.

### 6.1. Intrinsic Injury

This type of injury does not occur when the agent is given within at the approved therapeutic dose. As such, intrinsic injury occurs when there is an overdose of the drug or herbal product [10,15]. Hence, this type of injury is dose-dependent [10,20,50,51] and predictable [20,50,51] and has a short latency period [10,15]. Both DILI and HILI of this type can be reproducible in animal models [10,15]. Examples of plants that can cause this type of injury include plants that have alkaloids [15] such as bush tea (Crotalaria species) [15], Gynura segetum [15], and germander [15].

### 6.2. Idiosyncratic Injury

This type of injury is not dose-dependent [10,50,51], and as such, it is not predictable [15,50,51]; has latency periods that are quite variable, ranging from days to years [10]; and is not reproducible in animal models [10]. Nonetheless, it is the cause of most cases of DILI [50] and HILI [15,42]. From the etiological point of view, this type of injury can be caused by an immune or allergic reaction in a susceptive individual or can be a nonallergic and non-immunologic-mediated reaction [15,51]. However, in this type of injury, neither DILI nor HILI can be reproduced experimentally [10,15,42]. A prime example of this type of injury is that caused by the consumption of greater celandine [15].

## 7. Hepatotoxicity Initial Assessment

Liver toxicity is initially assessed by noticing if there are increases in the levels of liver enzymes above the upper limit of normal (ULN) of ALT and ALP [14,17,42,52]. However, the values beyond ULN that are deemed toxic differ between references.

The Council for International Organizations of Medical Sciences (CIOMS) defines hepatotoxicity as an increase in ALT and or ALP equal to or above 2 ULN [14].

The DILI Expert Working Group uses the same value of ALP as the CIOMS but defines the ALT to be either equal to or above 5 ULN or equal to or above 3 ULN with total bilirubin equal to or above 2 ULN to be considered hepatotoxic [14].

In other studies of HILI, including one by N. Quan et al., the following parameters were used to define hepatotoxicity: ALT or AST levels equal to or above 5 ULN, or ALP equal to or above 2 ULN, or serum levels of total bilirubin equal to or above 2 mg/dL and ALT equal to or above 3 ULN [52].

S. David and J.P. Hamilton considered ALP values above 2N diagnostic, while ALT had to be above 5N or 3N plus bilirubin values above 2N. Serum levels of ALT above 2N were not considered sufficiently diagnostic, because this would include many patients with increases due to other causes [42].

## 8. Liver Pattern of Injury

Both DILI and HILI can be classified as hepatocellular, cholestatic, or mixed, in terms of phenotypes or patterns of liver injury [14,15,17,20,51].

To make the distinction among the types mentioned above, it is necessary to calculate the R ratio [8,15,53,54]. The R ratio is estimated as (serum ALT value divided by its ULN)/(serum ALP value divided by its ULN) [8,53,54].

An R ratio above 5 means a hepatocellular injury, an R ratio below 2 means a cholestatic injury, and an R ratio between 2 and 5 means a mixed injury [8,14,15,53,54].

Hepatocellular is the most frequent type of injury, followed by mixed and cholestatic injury [17,55].

## 9. Risk Factors

Risk factors can be divided into host-dependent and natural product- or herb-dependent. A combination of factors determines not only the symptoms and signs presented by patients but HILI outcome [56]. In the case of DILI, pharmacological risk factors are well established; however, the same cannot be said for host-related risk factors [56]. We speculate that if what is mentioned above is true for DILI [56], it might be true for HILI as well.

### 9.1. Host Dependent Risk Factors

Host-dependent risk factors are related to the consumer of herbal products and include factors such as age, sex, alcohol consumption, and chronic liver disease [14,17,19,52,55,57].

In terms of age, it seems that HILI is more frequent in middle-aged patients, with a mean age of 45 years in several studies [17,19,52]. However, according to CIOMS, age as a risk factor is considered only in patients 55 years old or above [14].

In terms of alcohol consumption, according to CIOMS, patients at increased risk are those who consume more than two drinks per day, for women, or more than three drinks per day, for men [14].

Patients who develop HILI who also have a chronic liver disease have poorer outcomes when compared with patients that do not have a chronic liver disease [19].

In terms of sex differences, studies have conflicted even in the same country. In China, it seems that HILI affects men more often than women [19]. In the USA, the opposite happens, with women being more frequently affected than men [19]. In Korea, one study pointed to a higher incidence in males than in females [57]. However, another study in Korea showed just the opposite trend, with females having higher HILI incidence [45]. It seems that sex, as a risk factor, is not as clear-cut as age or alcohol consumption.

### 9.2. Herb-Dependent Factors

These are dependent on the herb or mixture of herbs included in the final herbal product. They include quality issues related to how the herbs are grown, harvested, stored, and processed into the final product for sale. They also include dose-related issues, meaning the concentration of active compounds present in the final herbal product.

Quality is defined as the final product having the same therapeutic effects independently from which batch it is used [58]. Quality is affected by multiple factors, such as cultivation, harvesting, and manufacturing methods [57,58,59]. Producers must follow good agricultural practices and good manufacturing practices for herbal products to be safe and without quality issues [15,46,59,60].

Almost every part of a plant can be used to make a herbal product. These include leaves, the aerial part of the plant, flowers, fruits, bark, roots, and seeds [20,45], as can be seen in Table 1. Consequently, herbal products must have a clear description of which part of which plant was used for the product at hand [45,59,61].

Lastly, these products must not have impurities, chemical adulterants, or misidentifications of plants or plant parts [59,61]. Herbal products can be contaminated with toxic elements such as pesticides, mycotoxins, and heavy metals such as lead, mercury, cadmium, and arsenic [19,20]. On the other hand, herbal product companies sometimes add lead, mercury, arsenic, and cadmium, believing that these compounds increase the products’ effectiveness [15,20]. These adulterants, in most cases, are unlabeled [15]. Sometimes, the herb indicated on the label might not be present in the preparation because of wrong identification [19]. Some mixtures of herbs might contain toxic components that may cause liver damage [19]. Other herbal products may contain the correct plant but not the indicated part of the plant; this is considered an adulteration [45]. The aforementioned issues provide high risk to the health of consumers of herbal products [15]. In order for herbal products to be safe for consumers, producers must abide by good agricultural and manufacturing practices [15].

Cultivation-wise, several factors can have an effect on quality, such as plant variety, type of soil, and climate in the area of cultivation. All of these factors have effects on the concentration of chemical substances present in the plant tissue, including substances with therapeutic properties [44,46,58,60]. One also has to consider the effect of pests and the use of pesticides to control or prevent damage to crops [44,58,59,60]. The factors and issues mentioned previously affect the final concentration of the active compound in the plant of interest [58].

The manufacturing process of an herbal product has an impact on the quality of the finished product as well [44,46,58]. For example, raw materials used in the manufacturing process can either be fresh or dried plant material [58,62]. These raw materials can then be processed into powdered plant material, expressed plant juice, tinctures, essential oils, or plant extracts [58,62].

The extract can either be dry, liquid, or soft [58]. Soft extracts are more prone to bacterial colonization and lead to more unstable products; as such, most of them have been replaced by dry extracts [58]. Liquid extracts contain, aside from compounds found in plants, solvents used in the extraction process [58]. Dry extracts are made by evaporating the solvents used [58]. All of these different pathways to the finished product can affect the final quality of the product [58].

Therefore, herbal products’ quality is considered quite variable [19]. When herbal products have poor quality, it makes it harder to assess causality [61].

Dose as a risk factor, on the other hand, is uncertain [15]. However, as HILI can be classified as either intrinsic or idiosyncratic [15], it is within reason to presume that caution should be used when a high daily dose or high cumulative doses of herbal products known to cause intrinsic injury are referred by the patient.

### 9.3. Lipophilicity

Chemical compounds can be lipophilic or hydrophilic. Lipophilic molecules are known to have an effect on pharmacokinetics and toxicity [8]. The higher the degree of lipophilicity of a molecule, the higher the chances of it being hepatotoxic [8,56]. During the drug development process, molecules known to have a higher degree of lipophilicity are less likely to pass from phase I to phase II clinical trials [56]. Lipophilic molecules lead to the generation of higher amounts of reactive metabolites in the liver and result in a higher likelihood of injury [8]. Both lipophilicity and drug metabolism are independent risk factors for DILI [8]. The degree of lipophilicity of a molecule combined with its daily dose above 100 mg, known as the “rule of two”, could be used to predict the severity of DILI in a clinical setting [8,63]. The rule of two is used as a predictive tool, during the drug development process, for distinguishing drugs with hepatotoxicity potential [56]. However, the prognostic ability of the rule of two in a clinical setting has not been proven, and further research is needed [8,56,63,64].

## 10. Diagnosis

HILI is a diagnosis of exclusion [15,16,20]. Clinicians need to have a high degree of suspicion [20], as when faced with a liver-affecting disease without a defined cause. The patient’s context is important. For example, if a patient is healthy before starting to use the herbal medicine, and the patient’s health worsens after starting the herbal medicine but improves after cessation of use, there is a high probability of HILI [20]. If the timeframe between starting consumption of the herbal product and the time it starts showing alteration of liver chemical parameters or symptoms is between 5 and 90 days, then HILI has a high score on the CIOMS scale [14]. It has a low score if the timeframe is fewer than 5 or more than 90 days [14]. HILI can mimic several different liver diseases, both acute and chronic [20]. Unfortunately, so far there has not been a description of either a test, a biomarker, or a gold standard for the diagnosis of herb-induced liver injury [20]. As such, to diagnose HILI, one needs to know several elements to discard alternative causes for the symptoms and signs presented by the patient [15,20,59]. The elements necessary to make a diagnosis are the following: the latency period; the clinical presentation; the patient’s risk factors for the development of HILI; biochemistry alterations, especially in liver enzymes (ALT and ALP); the patient’s medication; and the course of recovery (and sometimes rechallenge). A thorough search for other causes of liver diseases is also necessary, such as: viral parameters including the various hepatotropic viruses, HAV, HBV, HCV, HDV, HEV, Epstein–Barr virus (EBV), cytomegalovirus (CMV), herpes simplex virus (HSV), and varicella-zoster virus (VZV); autoimmune diseases; hepatic vascular diseases such as Budd–Chiari syndrome and sinusoidal obstruction syndrome; biliary diseases such as biliary obstruction, either intra- or extrahepatic; hepatic tumors; nonalcoholic fatty liver disease (NAFLD); nonalcoholic steatohepatitis (NASH); and inherited metabolic liver diseases such as hemochromatosis, Wilson’s disease, and Alpha1-antitrypsin deficiency [14,15,20,59].

### 10.1. Symptoms

Symptoms presented by HILI patients are very similar to symptoms presented by DILI patients [16,42]. Symptoms of both entities are considered unspecific, and the degree of variability between patients is substantial [15,42], as can be seen in Table 1. Clinically, patients harboring HILI can be asymptomatic, presenting with only increases in levels of liver enzymes; monosymptomatic; or polysymptomatic [20,42,49]. Symptoms presented by HILI patients are mainly intestinal, such as nausea, vomiting, and abdominal pain [20]. However, amid this variability, there is some degree of consistency. Following are some examples of symptoms presented by patients that used herbal products from traditional Chinese medicine or Ayurvedic medicine, as well as those containing greater celandine and pyrrolizidine alkaloids [42].

Patients that consumed traditional Chinese medicine herbal products exhibit symptoms of hepatotoxicity in the following decreasing order of frequency: fatigue, jaundice, anorexia, nausea, fever, rash, pruritus, and pale stools [15,42]. HILI in these patients usually presents with a slow symptomatic evolution, having a long latency time [42].

In the case of Ayurvedic herbal products, the symptoms tend to follow a stepwise pattern, starting with pruritus and followed by loss of appetite, fatigue, nausea, vomiting, dark urine, light stool, and jaundice [15,42].

Another example of hepatotoxicity is that presented by greater celandine. This plant causes symptoms of hepatotoxicity including jaundice, nausea, fatigue, anorexia, dark urine, pruritus, vomiting, dyspepsia, bloating, abdominal discomfort, right upper quadrant pain, pale stools, and fever [15,42].

Pyrrolizidine alkaloid (PA) is the name of a compound that is present in certain herbs [42]. PAs are known to be hepatotoxic in humans and are hepatocarcinogenic in mice [65]. PA causes a very distinct entity called hepatic sinusoidal obstruction syndrome [15,42]. Symptoms are abdominal distention and pain, ascites, malaise, hepatomegaly, increased body weight, and jaundice [15,42]. This type of injury is classified as intrinsic [42]. Furthermore, PAs are highly lipophilic molecules and can cross the placenta and cause embryotoxicity. As such, their use is recommended neither during pregnancy nor during the lactation period [42,66].

Some HILI cases, although rare, can evolve to acute liver failure, requiring emergency liver transplantation [15].

### 10.2. Biopsy

Liver biopsy is not a procedure used routinely for HILI management and diagnosis [20]. It is also not necessary for the Roussel Uclaf Causality Assessment Method (RUCAM) for either DILI or HILI [19]. This is because changes caused by drugs or herbs are not specific for either HILI or DILI [19]. Histopathological changes identified by the biopsy of patients with HILI are either hepatitis, cholestasis, fibrosis, the presence of eosinophil infiltrates, or necrosis [20]. For the reasons mentioned above, biopsy is not beneficial for the patient, because it does not help the clinician managing the patient's symptoms and is not exempt from complications [19,20].

### 10.3. Causality Assessment

Assessment of causality of HILI is very similar to that of DILI [15,67]. Both DILI and HILI are difficult and complex to diagnose and are often misdiagnosed. For the clinician to make the diagnosis of either accurately, it is crucial to have good clinical judgment and an assessment algorithm [14,67]. The CIOMS scale seems to be the preferred tool to assess causality worldwide, given that it is a validated, liver-specific, structured, and qualitative method [67].

There exist several different scales that attempt to measure the likelihood of HILI. Such scales are the RUCAM/CIOMS scale, the scale of Maria and Victorino, and the TTK scale, named after its authors, Takikawa, Takamori, and Kumagi. The TTK scale is used primarily in Japan [14,15,20,59].

The CIOMS/RUCAM scale is a method to predict the likelihood of liver damage caused by suspected drugs [68]. The RUCAM scale, also known as the CIOMS scale [68,69] was first published in 1993 and updated in 2016 [70,71,72,73]. This scale can be used for both DILI and HILI [72]. It is the most widely used scale worldwide [70,71,72,73]. It is a quantitative and, for the most part, objective scale [68,72].

The RUCAM scale is more accurate if used prospectively for acute liver injury and for idiosyncratic reactions. It has to be calculated for each suspected drug [68].

The RUCAM scale assigns different weights to several domains, such as latency time, risk factors, exclusion of other causes of liver disease, etc. [68,70,72,74]. After the clinician has summed up all the points, the scale gives a total score that is between 0 and 8. This total score categorizes the likelihood of causality as definite or highly probable (>8), probable (between 6 and 8), possible (3–5), unlikely (1–2), or excluded (≤0) [70].

The RUCAM scale uses different algorithms for hepatocellular and cholestatic or mixed injury [70,73]. Worksheets for both were available in the articles by Teschke [73] and Danan [68].

### 10.4. LiverTox

LiverTox is an interactive website that provides up-to-date, evidence-based, comprehensive, and unbiased information about DILI, prescription drugs, over-the-counter drugs, and herbs and food supplements [75,76]. LiverTox is a tool that can be used by clinicians, researchers, and the general public [76]. As the website writing is clear, without too many abbreviations or highly technical terminology, it is adequate for the general public interested in possible side effects of drugs and herbs [75].

On LiverTox, each part concerning a single chemical agent has eight sections: an introduction, background, hepatotoxicity, mechanism of liver disease, outcome and management, case reports, chemical, and product information, and references [75].

## 11. Treatment

The most effective treatment for HILI is for the patient to stop taking the suspected herbal product; this is followed by continuous close monitoring of the patient until symptoms and biochemistry parameters resolve [20,42]. Other treatments might include the use of glycyrrhizin, ursodeoxycholic acid, and corticosteroids [20,42]. Patients with hepatic sinusoidal obstruction syndrome can also benefit from the use of anticoagulation therapy [77]. Fulminant hepatitis can be serious enough to require a liver transplant [20]. For most cases of HILI, the patient recovers [20].

## 12. Prognosis

If HILI is diagnosed and the herbal product identified and its use stopped, in general, patients have a good outcome and prognosis [17,20,42]. Most patients experience an acute type of liver injury that normalizes once the consumption of the suspected herb is discontinued [20,42]. A study by R. Teschke et al. [61] showed that of their population, 81.8% had their biochemistry normalized, 14.1% ended up developing chronic HILI, 7.6% developed cirrhosis, 0.2% had to proceed to liver transplantation, and 3.9% died. Acute liver failure is a rare occurrence [19,42]. However, HILI patients that develop acute liver failure are transplanted more often [19]. The outcome is overall better for patients that do not have chronic liver diseases [19]. For patients diagnosed with hepatic sinusoidal obstruction syndrome, about half recovered both clinically and at the biochemistry level, 30% developed decompensated liver disease, and 20% died [78].

## 13. Table of Phytotherapy Products

Globalization has changed the world. As an example, TM with its origin in one region of the world can today be used anywhere and by anyone. As such, today, the clinician needs to be aware that HILI presented by patients can be due to herbs from different cultural backgrounds. This means that HILI is a worldwide issue.

As such, Table 1 is a compiled table with different herbs and herb products from different cultural backgrounds.

Table 1 shows some examples of herbs and herbal products that might be consumed by HILI patients. It provides information regarding the plant’s or product’s name, which plant part(s) is used, therapeutic indication, and toxicity.

## 14. Conclusions

Across the globe, there has been an increase in the use of herbal medicines, and with it, an increase in HILI cases. HILI diagnosis is complex. The clinician needs to have a high degree of suspicion, making HILI diagnosis particularly challenging. Both HILI and DILI are diagnoses of exclusion, meaning that they must be part of the differential diagnosis when an elevation of liver enzymes occurs without a proper explanation. Patients should be routinely inquired about their consumption of herbal products. Healthcare-related professionals need to be aware of the possibility of herbal products' associated risks and side effects. Cases of HILI should be reported to national pharmacovigilance systems, as they allow the understanding of possible causal relationships between herbs and their phytochemistry compounds and clinical outcomes, in this case, involving liver disease.

Difficulties in HILI diagnosis pertain to the perception of herbal products as safe. Hence, patients do not tell their doctors that they have used herbal products, which delays the correct diagnosis and might lead to complications. To decrease the incidence of HILI, in general practice settings, clinicians should inquire about patients’ awareness of possible risks of toxicity from consumption of herbal products and increase patients’ information related to the issue.

Our understanding of HILI would benefit greatly if more studies were conducted concerning the chemical substances that are present in herbs and their properties.

Further studies of HILI are needed to better understand its complexity and prevent increased morbidity and mortality.

## Figures and Tables

**Table 1:** A few examples of herbs and other phytotherapies with therapeutic intent from different parts of the world that can be encountered in a clinical setting.

Latin Name	Plant Part(s) Used	Therapeutic Use	Toxicity	References
*Actaea Racemosa L.*	Rhizomes and roots	Menopause symptoms, dysmenorrhea	Tremors, blood hypotension, vomiting, and hepatotoxicity	[23,24,25,26]
*Agathosma betulina*	Leaves	Diuretic, arthritis, cystitis, diarrhea	Gastrointestinal irritation and centrilobular hepatic necrosis	[26,27,28,29]
*Arctostaphylos Uva-Ursi L.*	Leaves	Urinary infection	Nausea and vomiting	[23,24]
*Artemisia argyi*		Malaria, hepatitis	Hepatocellular	[15,30]
*Borago Officinalis*	Flowers, leaves, and oil obtained from seeds	Skin problems	Possible hepatotoxicity and cancer	[23,24]
*Callilepis laureola*	Root	Gastrointestinal symptoms, impotence	Abdominal pain, vomiting, diarrhea, and liver failure	[26,28,29]
*Camellia sinensis*	Leaves	Antiulcerous, antihypertensive, antiinfection	Hepatocellular hepatitis, cholestatic hepatitis, and fulminant hepatitis	[25,26,27]
*Cassia angustifolia*		Laxative agent	Acute hepatitis	[25,26]
*Catha edulis*	Leaves	Obesity, depression	Hepatotoxicity, hepatitis, and duodenal ulcer	[26,28,29]
*Catharanthus roseus*	Leaves	Cancer chemotherapy	Medullary aplasia, leukopenia, and constipation	[26,27,28,29,31,32]
*Chelidonium majus*		Ulcer treatment, skin diseases, expectorant	Cholestatic type	[25,26,33,34]
*Cordia salicifolia*		Diuretic, weight reduction	Acute hepatitis and hepatocellular hepatitis	[25,26]
*Crotalaria laburnifolia*		Dysmenorrhea, abortion induction	Hepatic veno-occlusive disease	[26,28,32,35]
*Croton cajucara Benth*		Obesity and hypercholesterolemia	Acute, chronic, and fulminant hepatitis; cholestatic type	[25,26]
*Dichroa febrifuga*	Roots	Malaria, cancer, fibrosis, inflammatory diseases	Hepatocellular	[30]
*Ephedra sinica*	Aerial plant part	Weight reduction	Acute hepatitis, autoimmune hepatitis	[15,36]
*Equisetum*		Abdominal pain, obesity, antipyretic	Chronic hepatitis, cirrhosis	[25,26,31,33,37]
*Feruca Asa Foetida L.*	Oil and resin from roots	Gastritis, duodenitis, colitis, irritable bowel syndrome	Lip dryness and edema, flatulence, diarrhea, headache, and malaise	[23,24]
*Larrea tridentata*		Common cold, HIV infection	Acute and fulminant hepatitis, cholestatic type, and cirrhosis	[25,26,35,38]
*Mentha Pulegium L.*	Flowers and essential oil	Coryza, dyspepsia, external use for skin diseases	Hepatotoxicity due to essential oil when used in high doses or for long periods	[23,24,25,26,33,37]
*Pelargonium sidoides*	Rhizome	Pulmonary tract symptoms of infection or irritation, gastrointestinal tract symptoms, pain	Nausea, heartburn, and diarrhea	[26,28,29]
*Petroselinum Crispum*	Fruits and roots	Prevention of urinary infection, kidney lithiasis	Essential oil neurotoxicity, abortive. High therapeutic doses cause hepatic and kidney lesions and arrhythmias	[23,24]
*Phyllanthus Amarus Schum*	Leaves and aerial parts	Chronic hepatitis, urinary lithiasis	Pirrolizidinic alkaloids present	[23,24]
*Piper Methysticum Forst. Fil*	Rhizomes	Anxiety, stress, insomnia	Hepatotoxicity and hyperbilirubinemia	[23,24,25,39]
*Plantago ovata*		Laxative agent	Acute hepatitis	[25,26]
*Prunus africana*	Bark	Prostate-related disorders, urinary infections	Gastrointestinal symptoms	[26,27,28,29]
*Pulmonaria Officinalis L.*	Flowers	Lung diseases	Hepatotoxicity when high therapeutic doses are used for long periods	[23,24]
*Rhamnus purshiana*		Laxative agent	Acute and chronic hepatitis and cholestatic type	[25,33]
*Ricinus communis*		Ulcers, cancers, tumors, warts	Hepatocellular	[30]
*Rumex Crispus L.*	Rhizomes, roots, and leaves	Anemia, upper airway inflammation	Oxalic intoxication, hypokalemia, metabolic acidosis, and hepatic and kidney failure	[23,24]
*Securidaca longepedunculata*	Roots and bark	Laxative, coughs, headaches, fever	Diarrhea and acute interstitial nephritis	[26,27,28,29]
*Serenoa Repens*	Fruits	Benign prostatic hyperplasia	Diarrhea, constipation, skin reactions, and cholestatic hepatitis	[23,24,35,38]
*Symphytum Officinale L.*	Roots	Topical use for sprains, bruises, luxations, fractures	Hepatotoxicity	[23,24,40]
*Tussilago Farfara L.*	Flowers and leaves	Airway inflammation, mouth and pharynx mucosa inflammation, bronchitis, asthma, emphysema	Hepatotoxicity due to pyrrolizidinic alkaloids	[23,24]
*Uncaria tomentosa*		Arthritis, back pain, gout	Acute hepatocellular hepatitis	[25,26]
*Vaccinium Myrtillus L.*	Fruits and leaves	Diabetes mellitus, microcirculation problems	Worsening of gastritis and gastroduodenal ulcer. High doses and prolonged use of leaves caused jaundice in laboratory animals	[23,24]
*Vaccinium Vites Idaea L.*	Leaves	Urinary infection	Prolonged use may lead to hepatotoxicity	[23,24]
*Valeriana officinallis*		Insomnia, anxiety, stress.	Acute and fulminant hepatitis	[25,26,33,37]
*Dai saiko-to*	Several different parts	Immunostimulant	Autoimmune hepatitis	[15,36,41]
*Jin Bu Huan*	Several different parts	Sedative	Acute and chronic hepatitis and fibrosis	[15,36,41]
*Paeonia spp.*	Several different parts	Atopic dermatitis	Acute hepatitis and fulminant hepatic failure	[36]
*Sho-saiko-to*	Several different parts	Chronic liver diseases	Acute and chronic hepatitis	[36]

White spaces mean no data.
